# Academic Burnout, Family Functionality, Perceived Social Support and Coping among Graduate Students during the COVID-19 Pandemic

**DOI:** 10.3390/ijerph20064832

**Published:** 2023-03-09

**Authors:** Diego Andrade, Icaro J. S. Ribeiro, Viktória Prémusz, Orsolya Maté

**Affiliations:** 1 Faculty of Health Sciences, University of Pécs, 7621 Pécs, Hungary; 2 Nursing Faculty, Southwest Bahia State University, Jequié 45083-900, Brazil

**Keywords:** burnout syndrome, coronavirus pandemic, student burnout, learning burnout, doctoral student, master’s student, mental health, social network, family functionality

## Abstract

Academic burnout and the COVID-19 pandemic have greatly impacted the academic life and mental health of graduate students. This study aims to address the mental health issue in graduate students by relating it to family functionality, perceived social support, and coping with academic burnout during the COVID-19 pandemic. Data were gathered from a cross-sectional study with 519 graduate students across universities in Hungary and other European countries. The Copenhagen Burnout Inventory Student, Family APGAR Index, the brief form of the Perceived Social Support Questionnaire, and the Brief Resilient Coping Scale were used to measure academic burnout, family functionality, perceived social support, and coping, respectively. Structural equations modeling was used for statistical analysis. The results revealed a negative effect of family functionality, perceived social support, and coping on academic burnout. The inverse relationship between perceived social support and the Copenhagen Burnout Inventory was identified, and mediated by coping and family functionality. These findings may offer patterns and predictors for future graduate students and higher-education institutions to identify outside factors that are implicated in academic burnout, especially in outbreaks such as the COVID-19 pandemic.

## 1. Introduction

Academic burnout can be defined as an exhaustion resulting from the excessive and prolonged stress experienced in academia [1]. The development of burnout in students is directly related to academic overload with numerous deadlines and assignments, and it is associated with drained energy, reduced enthusiasm toward academic tasks, lack of positive attitudes, and low academic achievements [2,3,4,5]. Burnout in students has been shown to harm the mental health of students and it is increasing at a pandemic-like scale among graduate students [6,7,8].

Graduate students are additionally dealing with another pandemic, the unpredictable COVID-19. Governments worldwide imposed restrictive measures to prevent the spread of SARS-CoV-2 virus. Hygiene measures (i.e., masks, hand disinfection), and social distancing (i.e., lockdown, physical distancing, quarantines) were adopted [9]. Higher education institutions were forced to quickly adapt from conventional learning to online learning, and students have had to deal with online learning challenges [10]. Online learning indeed helps prevent the spread of COVID-19, but also contributes to worsening the effects on mental health in graduate students [11,12]. Conducting research, studying, and dealing with other academic demands in these stressful environments can result in long-term psychological consequences and trigger academic burnout. Studies have shown that graduate students are more affected by mental health issues and are more likely to present increased stress levels compared to undergraduate students and the general population during COVID-19 [13,14].

The COVID-19 pandemic negatively impacted the mental health of students, and studies have been demonstrating increased distress and detrimental burnout symptoms among graduate students during the lockdown. Associations with low engagement, low motivation, poor work-life balance, and academic dissatisfaction were found among graduate students who scored with moderate to high levels of academic burnout. Numerous factors were cited by the participants, such as increased workload, uncertainty about the future, fewer learning/job opportunities, research prolongation, funding/grant discontinuation, lack of respect and understanding from supervisors, inadequate mentoring, reduction of concentration in online learning, and fatigue [15,16,17,18].

Although burnout is known as a work environmental syndrome, factors outside academia may also influence burnout. Within the literature, social support is one of the concepts that contributes the most to academic burnout. Perceived social support is how individuals perceive they are cared for by friends, family members, and others, especially during times of need. Perceived social support has important benefits in mental health since it is a significant predictor of life satisfaction and well-being [19,20].

The absence of social support increases chronic stress (i.e., a burnout predictor), and its presence decreases academic burnout [21,22]. A supportive network can help the student in developing effective coping strategies. Coping can be defined as cognitive and behavioral strategies used in response to stressful events [23]. Effective coping might reduce the impact of stressful situations on physical and mental health by building strategies to shield against academic burnout [13,24,25]. Otherwise, unhealthy or poor coping strategies can result in higher levels of stress and increased burnout [26,27].

Within the social network, family plays an important role in academic burnout, as cited by previous studies. The family relationship is a role of co-responsibility among the members and constitutes an important source of care and affection and the development of values, behaviors, and other life skills for the stress management of each family member [28,29]. Significant associations between burnout and family relationships have been reported by previous studies, and a difficult family environment can lead to academic burnout, including increased stress due to decreased family functionality and work/family management [30,31,32].

The influence of multiple factors, such as family, social support, coping, and the usual academic environment can create pressure on students and play important mediating roles in academic burnout. Specifically, studies have found that students who reported higher levels of social and family support were more likely to use active coping strategies, such as seeking social support and problem-solving, which in turn was associated with lower levels of academic burnout [22,24,32].

In the context of graduate students, this pressure eventually depletes emotional and mental resources, resulting in the detriment of mental health and academic burnout. Based on these findings, it is clear that burnout is a serious problem and may negatively impact the meaning and value of the respective degree for the graduate student, with consequent mental health issues. This study addresses a critical research gap concerning mental health and academic burnout among graduate students by relating it to family functionality, perceived social support and coping during the recent COVID-19 pandemic.

Considering that the physical environment of academia itself it is restrained due to curfews, quarantines, restrictions on social interaction, and the switch to online learning, we questioned the influence of family functionality, social support and coping among graduate students with regard to burnout syndrome during the COVID-19 pandemic. We hypothesize that graduate students experienced greatly worsened burnout during the COVID-19 pandemic when dysfunctional families, low perceived social support and poor coping strategies were present. We aim to address the mental health issue in graduate students by relating it to family functionality, perceived social support, and coping with academic burnout during the COVID-19 pandemic.

## 2. Materials and Methods

### 2.1. Study Design and Data Collection 

This is a cross-sectional analytical study. Data were collected through an online questionnaire between September 2021 and March 2022. The questionnaire was distributed virtually through the Google Forms platform, in close cooperation with international associations of graduate students and university departments. Participants were recruited through emails, social media channels of communities for graduate students, and through referrals from eligible participants. On the welcome page of the online questionnaire, participants were informed about the research and the informed consent was obtained from all those agreeing to answer the survey by clicking the ‘I consent’ button at the bottom of this same page. It was displayed that the survey would take approximately 15 min to complete.

Participation was anonymous and voluntary throughout the entire study period, and participants were informed about the research and its goal before giving their consent. We were unable to assess how many people viewed the online invitation, and therefore we could not determine participation rate of the study. Altogether, 542 students participated in the study. After eliminating incomplete answers, the final sample consisted of 519 graduate students which yielded a 95.75% completion rate.

The inclusion criteria were being a graduate student at master’s or PhD/DLA level, fluent in English, and voluntarily participation. Exclusion criteria were incomplete questionnaires and those who did not wish to participate in the research. Incomplete questionnaires with missing responses were excluded from the study.

### 2.2. Measures and Variables 

The questionnaire used in this study consisted of four sections, namely Copenhagen Burnout Inventory—Student version (CBI-S) [33], Family APGAR Index (Family APGAR) [34], the brief form of the Perceived Social Support Questionnaire (F-SozU K-6) [35,36] and the Brief Resilient Coping Scale (BRCS) [37]. All questionnaires have a psychometric content that quantitatively assessed traits linked to the psychological functioning of the evaluated individuals.

The CBI was developed by Kristensen et al., and adapted for students by Campos, Carlotto, and Maroco [38]. This scale consists of 25 items that represent 4 subscales: Personal Burnout (PB), Studies-related Burnout (SRB), Colleague-related Burnout (CRB), and Teacher-related Burnout (TRB). It is a 5-point Likert scale, ranging from 1 (never) to 5 (always). The answers are quantified as 0, 25, 50, 75, and 100% respectively, with a reverse scoring for item 10. We used the Kristensen’s criteria for burnout score: 50 to 74 is considered moderate, 75–99 is high, and a score of 100 is considered severe burnout [39]. In the current study, the Cronbach’s alpha for the CBI-S scale was 0.93, indicating good internal reliability.

The Family APGAR, consisting of five questions, was developed by Smilkstein [34] and has well-established reliability and validity. This scale evaluates a family member’s perception of family functioning by assessing his/her level of satisfaction with each statement on a 3-point Likert scale, ranging from 0 (hardly ever) to 2 (almost always). The points from each item were calculated to obtain the total score. A higher score indicated better family functioning [28,40]. The Family APGAR scale has been widely used, with satisfactory reliability and validity [28,41]. In the current study, Cronbach’s alpha value was 0.87.

The brief version of F-SozU K-6, developed by Kliem et al. [36], measures general perceived social support, rated on a 5-point scale ranging from 1 (not true at all) to 5 (very true). Higher scores indicate higher levels of perceived social support. The Brief Perceived Social Support Questionnaire has been widely used, with satisfactory reliability and validity [35]. In the current study, Cronbach’s alpha value was 0.86.

The BRCS was developed by Sinclair and Wallston [37] and is a measurement tool that has been proven to measure resilience with adequate levels of reliability and validity. BRCS has a unidimensional outcome conceptualized to assess the ability to handle stress in a highly adaptive manner. It is a 4-item scale with five options, where 1 means the statement “does not describe me at all” and 5 means “it describes me very well”. The sum score ranges from 4 to 20; the higher the score, the more resilient. The BRCS scale has been widely used, with satisfactory reliability and validity [42]. In the current study, Cronbach’s alpha value was 0.78.

### 2.3. Statistical Analysis

We considered the potential influence of academic burnout on the loss of family functionality, social support, and coping while constructing the theoretical model. We used structural equations modeling (SEM) to identify the effect between the measurement scales of these variables (i.e., academic burnout and Family APGAR, social support and coping). SEM consists of analyzing trajectories, characterized by addressing the problems of dependence between variables. The proposed structural equation model included all observable variables directly and indirectly (i.e., latent variable). The measurement model (confirmatory factorial analysis) was performed for CBI as a latent variable, using each of the components of the instrument in question as indicators. The other variables were measured directly and their relationships measured by multiple regressions. A *p*-value of 0.05 (two-tailed) was considered as statistically significant.

To assess the model fit, the root mean square error of approximation (RMSEA) was used. Values lower than 0.05 indicated adequate fit, with an upper limit of the 90% confidence interval lower than 0.08 [43]; the comparative fit index (CFI) and the Tucker–Lewis index (TLI) with values above or equal to 0.95 indicated a good fit; and, the standardized root mean square residual (SRMR) with a value of less than 0.05, was considered a good fit [43,44].

Standardized coefficients (SC) were interpreted according to Kline [43], where an SC of 0.10 indicates a small effect, an SC of 0.30 indicates a medium effect, and an SC > 0.50 indicates a strong effect.

Mplus software, version 7 (Muthén & Muthén, Los Angeles, CA, United States) [45] was used for the statistical analysis. The estimation was performed using the mean-corrected Satorra–Bentler’s maximum likelihood method (MLM), due to the absence of univariate and multivariate normality.

## 3. Results

A total of 519 graduate students who participated in the study (365 women [70.30%]) with a mean age of 31 years (±7.76) were evaluated. Overall, 292 (56.30%) were doctoral students enrolled in PhD or DLA programs and 227 (43.70) were students studying at master’s level. Of all graduate students, 360 (69.40%) were in the first and second year, 116 (22.40%) in the third and fourth year, and 43 (8.30%) in the fifth year or beyond of their degree. Characteristics of the study population is shown in Table 1.

**Figure 1:**
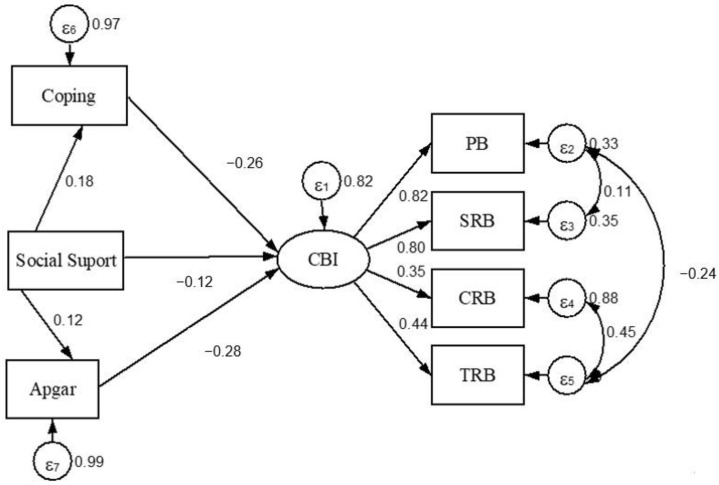
Visual representation of the structural model. CBI: Copenhagen Burnout Inventory-Student; PB: Personal Burnout; SRB: Studies-related Burnout; CRB: Colleague-related Burnout; TRB: Teacher-related Burnout.

The structural model proposed below (Figure 1) shows the totality of the proposed relationships. The standardized effects are presented in Table 2. The discriminant validity of the variables involved could be attested, since none of the correlations were greater than 0.9. Furthermore, it was found that the proposed model presented better indices of model adjustment: CFI = 0.98; TLI = 0.96; RMSEA (90% CI) = 0.049 (0.04–0.07); and SRMR = 0.02.

The measurement component analysis shows that the factor loadings (FC) were significant for the latent variable CBI (PB = 0.82; SRB = 0.80; CRB = 0.40; TRB = 0.50). Despite the CRB indicator being below the recommended cutoff (0.5), it was maintained in the model to respect the validated version of the proposed construct. It was then possible to provide evidence of the presence of a greater contribution of the PB and SRB indicators in the composition of the latent CBI (Table 2).

The observation of the structural model (Figure 1) showed a moderate and negative direct effect of Family APGAR (−0.28) and coping (−0.26) on burnout measured by the CBI-S. Furthermore, a weak negative effect of social support (−0.12) on burnout was also found. These findings provide evidence that an increase in family functionality, coping, and social support can reduce burnout level.

Upon observing the indirect paths of effect, it was identified that the inverse relationship between social support and CBI-S was mediated by coping (−0.05) and Family APGAR (−0.03), even though this path had a small but statistically significant effect.

## 4. Discussion

The current study discloses the influence of academic burnout on the mental health of graduate students by relating it to family functionality, perceived social support, and coping during the COVID-19 pandemic. We revealed a negative effect of family functionality, perceived social support, and coping on academic burnout. The opposite direction was also observed; the higher the family functionality, perceived social support and coping, the lower the academic burnout. Among the academic burnout dimensions, personal burnout and studies-related burnout were the ones that contributed most to the composition of the latent CBI-S.

We found a negative effect of family functionality on academic burnout as measured by the Family APGAR Index. This complex and dynamic relationship present in most families can be healthy and functional when the members live in harmony, and protects the integrity and the functional autonomy of the family system. Unhealthy and dysfunctional dynamics occur when there is a lack of compromise between the members. This is primarily caused by prioritizing interests that are detrimental to the other members [28,29].

Reports of the association between academic burnout and family functionality are limited [30,46], and to the best of our knowledge, our study is the first to explore this relationship among graduate students during COVID-19. Graduate students have to deal with their work–life balance, especially between their academic life and their families. While pursuing a master’s or doctorate degree, a great workload with considerable productivity is expected, and this may contribute to family issues and expose the students to burnout [7,47]. The results of our study suggested that this finding also applied to graduate students during the COVID-19 pandemic.

Curfews, restricted traveling, and quarantines forced families to remain in their homes. Due to this social isolation and confinement, students experienced new demands in addition to their preexistent roles in the family, such as online learning, managing a home office, fear of being infected and exposing their family, financial concerns from funding/grant discontinuation, and many others. This resulted in exhaustion and family imbalance, which can sustain family cohesion and support the social network system or generate a family crisis by creating new difficulties, dissatisfaction, and violence in some cases, affecting all the family members [15,16,48,49,50]. This association shows that the mandatory COVID-19 isolation forced families to spend more time together. This interaction may expose more family issues and problematic relationships, which can affect the family environment/functionality and further expose the graduate student to academic burnout.

The perceived social support in this study refers to the graduate student’s overall impressions of others regarding their availability and effective help when the student is in need [51]. When this social network works, the help is usually offered by family members, friends, colleagues, supervisor, and teachers by either material resources and/or psychological support [52,53,54]. Social support, therefore, helps to regulate the stress itself, as well as the impact on the individual coping process, by providing a better way to deal with the issues [55].

Social support has been shown to be negatively associated with academic burnout and correlated with low academic engagement and loneliness, even before the COVID-19 pandemic. The main social support predictors of burnout include insufficient support from supervisor/research community and especially from friends, low participation in extracurricular activities, and lack of leisure [56,57,58]; these factors were worse during the COVID-19 pandemic, when loneliness became more frequent and support from friends and the academic community shifted predominantly to online chats and meetings. In our finding, social support was the lowest in the construct. Social support from family members may have contributed to this, along with the use of social media to connect and communicate with others experiencing similar situations. 

In line with other research, the reduction of social support in our study is linked with maladaptive coping strategies in stressful situations which directly affect the mental health of students and their study engagement and performance, all of which contribute to academic burnout [22,26,59,60,61]. Social support has a protective effect on academic burnout, since the perception of a good supportive network increases motivation and engagement in studies, and overall life satisfaction for the student [61,62]. Perceived social support has been linked with good coping strategies that help students to develop resilience and perseverance to achieve their goals, allowing students to be less prone to stress and consequently protecting them from academic burnout [54,59,63]. 

A functional family serves as a protective factor against academic burnout, and helps the student to develop proper autonomy and adequate problem-solving strategies. These may shape the student’s resilience to deal with university demands, difficult issues and academic pressure [46,64].

Our finding, which contribute the PB and SRB dimensions to the composition of the latent CBI, are in agreement with the report data presented in previously published studies [38,65]. PB is related to the level of physical and psychological exhaustion experienced by the graduate student, and SRB is related to the level of physical and psychological exhaustion perceived by the graduate student in association with the academic work and tasks [33]. These dimensions provide a clear indication of burnout [65]. These findings show that the personal stressors and those related to study contribute directly to graduate student exhaustion and academic burnout. 

As mentioned previously, our findings highlight the crucial role of family functionality, perceived social support, and coping in the mental health of graduate students as predictors of academic burnout, particularly in the context of the COVID-19 pandemic. Graduate students may benefit from this study by understanding the causes of particular behaviors related to academic burnout and by providing insight to higher education institutions on how to make improvements during online learning, especially in the midst of pandemics such as COVID-19. However, our study has some important limitations, including the cross-sectional design, which limited our ability to establish causality between the associations. To address this, future experiments or longitudinal studies may be more suitable. Furthermore, future researchers may consider the extension of this work by exploring other factors beyond academia that predict academic burnout. Additionally, the online assessment used to collect data during the COVID-19 outbreak may carry response bias, making them less reliable. Finally, the research was conducted during the COVID-19 pandemic and hence burnout levels may have been impacted by this. Therefore, we have used screening tools in this study and our findings should be interpreted carefully, since it is not a clinical psychiatric diagnostic instrument. 

## 5. Conclusions

This study analyzed predictive factors outside of academia that significantly influenced the development of academic burnout among graduate students during the COVID-19 pandemic. These factors are family functionality, perceived social support, and coping. Low perceived social support and poor coping results in a negative impact on academic burnout for graduate students, especially in dysfunctional family settings. In a functional family, with the perception of strong social support, and appropriate coping, the opposite outcome is expected. We believe that these findings can offer patterns and predictors for future graduate students and to higher education institutions to better identify external factors implicated in academic burnout, specifically in stressful settings such as a pandemic. It is important to mention that these adverse consequences are not only for the graduate students but also a concern for academia, since mental issues directly impact the quality and quantity of research. We suggest that academia implements improvements to its institutional support in order to prevent academic burnout among graduate students.

## Figures and Tables

**Table 1:** Characterization of evaluated graduate students.

	n	%
**Gender**		
Male	154	29.7
Female	365	70.3
**Marital Status**		
Single	287	55.3
Married	123	23.7
Other	109	21.0
**Housing Member**		
Family	187	36.0
Partner	144	27.7
Friends/flatmates	77	14.8
Alone	109	21.0
Other	2	0.4
**Students’ country of origin**		
Hungary	183	35.3
European Countries	111	21.4
Non-European Countries	225	43.4
**Current level of education**		
Master’s degree	292	56.3
PhD/DLA degree	227	43.7

**Table 2:** Standardized coefficient (SC), 95% confidence interval (95%CI), and *p*-value of the structural equation model.

	SC	*p*	95%CI
**Measurement model**			
Personal burnout (PB) ← CBI-S	0.82	<0.01	0.64–0.99
Studies-related burnout (SRB) ← CBI-S	0.80	<0.01	0.64–0.96
Colleague-related burnout (CRB) ← CBI-S	0.40	<0.01	0.24–0.45
Teacher-related burnout (TRB) ← CBI-S	0.50	<0.01	0.33–0.55
**Structural model**			
**Direct effect**			
CBI-S ← Social Support	−0.12	<0.05	−0.21–−0.02
CBI-S ← Coping	−0.26	<0.01	−0.37–−0.18
CBI-S ← Family APGAR	−0.28	<0.01	−0.36–−0.16
Coping ← Social Support	0.18	<0.01	0.09–0.26
Family APGAR ← Social Support	0.12	<0.01	0.04–0.20
**Indirect effect**			
CBI-S ← Coping ← Social Support	−0.05	<0.01	−0.07–−0.02
CBI-S ← Family APGAR ← Social Support	−0.03	<0.05	−0.06–−0.01

Note. CBI: Copenhagen Burnout Inventory-Student; PB: Personal Burnout; SRB: Studies-related Burnout; CRB: Colleague-related Burnout; TRB: Teacher-related Burnout.

## Data Availability

Data of this study are available upon request.
